# The relationship between hematological indices as indicators of inflammation and 25-hydroxyvitamin D3 status in newborns

**DOI:** 10.1186/s12887-023-03903-8

**Published:** 2023-02-18

**Authors:** Yusuf Elgormus, Omer Okuyan, Hafize Uzun

**Affiliations:** 1 Faculty of Medicine, Department of Pediatrics, Istanbul Atlas University, Medicine Hospital, Istanbul, 34408 Turkey; 2 Faculty of Medicine, Department of Medical Biochemistry, Istanbul Atlas University, Istanbul, Turkey

**Keywords:** Hematological indices, Lymphocyte to monocyte ratio, Neutrophil to lymphocyte ratio, Newborn, Platelet to lymphocyte ratio, Vitamin D status

## Abstract

**Background:**

There is still much unknown about the relationship between hematological parameters and vitamin D status in newborns. The aim of the study is to evaluate the relationship between 25(OH)D3 (vitamin D) status and new defined systemic inflammatory markers neutrophil to lymphocyte ratio (NLR), lymphocyte to monocyte ratio (LMR), and platelet to lymphocyte ratio (PLR) in newborns.

**Methods:**

One hundred newborns were enrolled in the study. Serum vitamin D status, below < 12 ng/mL (< 30 nmol/L) as deficient, 12–20 ng/mL (30–50 nmol/L) as insufficient, and > 20 ng/mL (> 50 nmol/L) was considered as sufficient.

**Results:**

Parallel to maternal and newborn vitamin D status were also statistically different between the groups (*p* < 0.05). Moreover, there was a statistically significant difference was found between the deficient, sufficient and insufficient groups in terms of newborn hemoglobin, neutrophil, monocytes, NLR, PLT, PLR and neutrophil to monocyte ratio (NMR) (*p* < 0.05, in all). There was also a positive correlation between maternal and newborn vitamin D status (*r* = 0.975, *p* = 0.000). The newborn NLR were negative correlated with newborn vitamin D status (*r* = -0.616, *p* = 0.000).

**Conclusions:**

The results of this study suggest that there may be potential new biomarkers to predict inflammation associated with the inflammatory state that may arise due to changes in NLR, LMR, and PLR in vitamin D deficiency in newborns. NLR and other hematologic indices may be non-invasive, simple, easily measurable, cost-effective markers of inflammation in newborns.

## Backround

Vitamin D is a fat-soluble vitamin that a group of steroids have hormone-like functions. Most of the circulating vitamin D is 25-hydroxy-D3 [25 (OH) D3] (vitamin D), also known as calcidiol, which has little biological activity, a half-life of 15–20 days and thus showing the storage state in the body. It is also the form measured in vitamin D status assessments [1]. Today, vitamin D deficiency is an important public health problem that has serious negative effects on health during life, including pregnancy and infancy. In recent years, intergenerational chronic disease risk of vitamin D deficiency has been emphasized. It has been observed that there is an increase in evidence-based data showing that the risk increases in many diseases such as type 1 DM, (T1DM), multiple sclerosis, rheumatoid arthritis, hypertension, cardiovascular diseases, schizophrenia, Crohn’s, tuberculosis and many types of cancer [2–8].

Complete blood count (CBC) is simple, inexpensive, but includes important follow-up parameters for many diseases. Red cell distribution width (RDW) is a measure of the distribution of erythrocytes in hemogram parameters depending on their diameter or volume. RDW is a coefficient of variation and is calculated by 1 standard deviation of the formula erythrocyte volume / mean corpuscular volume (MCV) × 100. The positive association of RDW levels with inflammatory processes, especially C-reactive protein (CRP) and erythrocyte sedimentation rate (ESR), has been demonstrated in large cohort studies [9]. Neutrophil lymphocyte ratio (NLR) and platelet lymphocyte ratio (PLR) is an inexpensive and easily calculable index that correlates with the prognosis of systemic inflammatory diseases. In addition, it is reported in the literature that, similar to vitamin D, NLR, PLR, lymphocyte to monocyte ratio (LMR) and mean platelet volume (MPV) may also be an indicator of systemic inflammation [10–12].

As mentioned above, the relationship between vitamin D deficiency and the inflammatory state is well established. Therefore, the hypothesis of the study was that vitamin D deficiency may have inflammatory effects on CBC parameters. There is no study showing the relationship between inflammatory hematologic indices and 25(OH)D3 (vitamin D) status in newborns in the literature review. The aim of the study is to evaluate the relationship between vitamin D status and new defined systemic inflammatory markers NLR, LMR, and PLR in newborns.

## Materials and methods

**Fig. 1:**
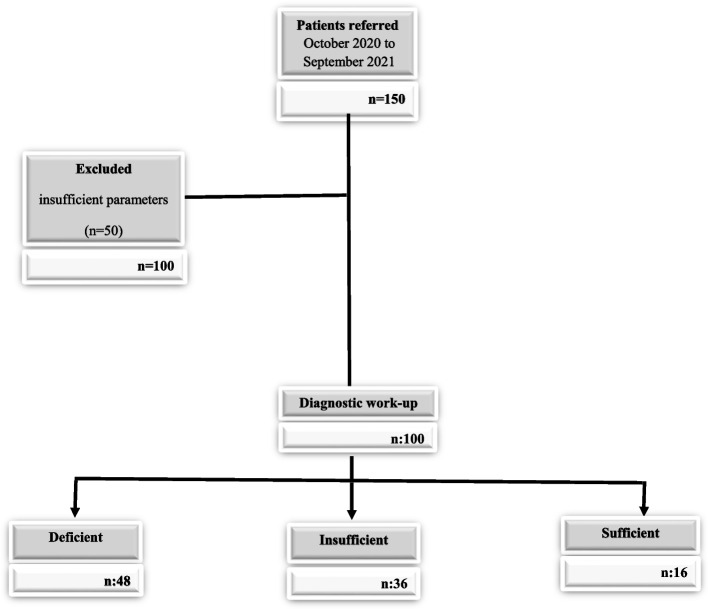
Flow diagram of the study process

This study was approved by the Istinye University ethics committee, Istanbul (Approval Date: September, 15, 2020; number: 82) and was conducted in accordance with the Declaration of Helsinki. Informed consent was obtained from legal guardian(s) of subjects. This was a retrospective study that conducted in the Medicine Hospital, Clinic of Pediatrics, İstanbul, Turkey. From October 2020 to September 2021, 100 newborns were enrolled in the study (Fig. 1).

Information about mothers and their babies’ demographic values (age and sex), weight, height, parity, socio-economic status, daily sun exposure, daily vitamin D intake, clothing style, season, serum vitamin D status, and hemogram results were recorded. All information was obtained from the subjects’ medical records. The mothers were divided into three groups according to daily vitamin D supplementation; none (0 vitamin D IU/day), irregular usage (approximately 400–600 IU/day), regular usage (approximately 1000–1200 vitamin D IU/day).

### Exclusion criteria

Newborns of mothers younger than 20 or over 40, mothers with chronic illnesses, mothers taking medication, having twin pregnancy and pregnancy with infections or inflammations. Patients with fetal inflammatory syndrome were also excluded from the study. Being small for gestational age (SGA; defined as a birth weight less than 2500 g), prematurity, having neonatal jaundice, having a congenital disease or malformation, age at sampling older than 1 week and refusal of parental consent were exclusion criteria for newborn.

Vitamin D levels were classified as follows; Serum 25(OH)D3 level below < 12 ng/mL (< 30 nmol/L) was considered as deficient, 12–20 ng/mL (30–50 nmol/L) as insufficient, and > 20 ng/mL (> 50 nmol/L) was considered as sufficient [13].

Blood samples for CBC and vitamin D were taken from the newborn within one week after delivery. Blood samples were taken into standardized tubes without anticoagulant and containing dipotassium ethylenedinitro tetraacetic acid (EDTA) for CBC parameters. The result of CBC was recorded with automatic hematology analyzer (Sysmex XN-1000, Norderstedt, Germany). NLR, LMR, and PLR were calculated from neutrophil/ lymphocyte/ monocyte/ thrombocyte count. The vitamin D levels were measured by enzyme linked fluorescent assay on the Mini Vidas (Biomerieux, Paris, France).

### Statistical analysis

Statistical analyzes of the data were analyzed with the SPSS Statistics (Version 26.0. Armonk, NY: IBM Corp.) package program. Parametric tests were used in the analysis of normally distributed data, and non-parametric tests were used in the analysis of non-normally distributed data. The non-parametric Kruskal–Wallis test was used for the comparison of three groups that did not fit the normal distribution, and the Mann–Whitney U test, which is a Post-Hoc test, was used to understand from which group and/or groups the difference originated. Spearman correlation analysis was performed in the analysis of bilateral relations between non-parametric variables, and values less than 0.75–1.00, 0.50–0.75, and 0.50–0.00 were interpreted as strongly, moderately and weakly correlated, respectively. Statistical significance was accepted as *p* < 0.05 in all evaluations.

## Results

Vitamin D status of newborns according to maternal characteristics was shown in Table 1. As shown in Table 2, the maternal mean age of the deficient group is 30.92 ± 6.52 years, the mean age of the insufficient group is 29.11 ± 5.51 years and the mean age of the sufficient group is 28.50 ± 6.87 years. There was no statistically significant difference between the groups in terms of mean age. Moreover, there were 19 boys and 29 girls in the deficient group; 12 boys and 24 girls in the insufficient group, and 8 boys and 8 girls in the sufficient group.

**Table 1:** Vitamin D status of newborns according to maternal characteristics

	**n (%)**	**25(OH)D**_**3**_** levels (ng/mL)**	**p**^**a**^
**All newborns**	100	13 (8–18)	
**Gender**			0.99
**Girl**	61 (61.0)	13 (8–18)	
**Boy**	39 (39.0)	13 (8–19)	
**Maternal age (years)**			0.13
**20–30**	53(53.0)	15 (10–19)	
** > = 30**	47(47.0)	11 (8–18)	
**Maternal socio-economic status**			0.92
**Low**	52 (52.0)	13 (8–19)	
**Moderate**	48 (48.0)	14 (8–18)	
**Maternal daily sunlight exposure**			0.58
**Yes**	44 (44.0)	12 (8–18)	
**No**	56 (56.0)	14 (9–19)	
**Delivery route**			0.24
**Vaginally**	25 (25.0)	15 (10–21)	
**C-section**	75 (75.0)	13 (8–18)	
**Vitamin D supplementation during pregnancy**			a^***^, b^***^c^***^
**None**	35 (35.0)	13 (8–23)	
**Irregular usage**	43 (43.0)	16 (8–39)	
**Regular usage**	22 (22.0)	22 (6–46)	

p^a^ = Mann Whitney U test

a: None vs irregular usage; b: None vs regular usage; c: Irregular vs regular usage

^*^p < 0.05, **p < 0.01, ***p < 0.001

**Table 2:** Vitamin D status, hemogram and indices values in maternal and newborns

	**Deficient** **(*n* = 48)**	**Insufficient** **(*n* = 36)**	**Sufficient** **(*n* = 16)**
***Maternal***			
**25(OH)D3 (ng/mL)**	10 (8–12)	18(13–28)^a***,c***^	37(22–55)^a***,b***^
**Leukocyte (10**^**3**^**/mm**^**3**^**)**	7500 ± 1739	6800 ± 1292^a*^	6689 ± 1382
**Hemoglobin (g/dL)**	10,5(8,9–12,4)	9,6(7,7–10,5)^a***,c***^	10.7(9.6–11.7)^b***^
**Eosinophil (10**^**3**^**µL)**	0,14(0,10–0,60)	0,20(0,10–0,80)^a*^	0.15(0.10–0.40)
**Neutrophil (10**^**3**^**/mm**^**3**^**)**	4.2(0.8–9.7)	3.9(1.8–5.5)	3.7(1.3–5.2)
**Lymphocyte (10**^**3**^**/mm**^**3**^**)**	2,1(1,3–3.6)	2.2(0.6–3.8)	2.1(1.0–3.7)
**Monocyte (10**^**3**^**/mm**^**3**^**)**	0.56(0.20–1.70)	0.45(0.20–1.10)^a*^	0.53(0.20–1.00)
**Platelet (mm**^**3**^**)**	245(170–380)	320(220–470)^a***,c***^	223(200–235)^a**,b***^
**NLR**	1.9(0.6–2.7)	2.0(0.7–3.2)	1.9(0.7–3.0)
**LMR**	4(2–7)	5(2–11)^a***^	4(3–6)^b*^
**PLR**	117(100–138)	198(59–633)^a***c**^	110(81–149)^b**^
**NMR**	7.4(3.7–9.1)	11.0(3.2–40.0)^a***,b**^	8.5(6.3–12.5)^a***,c**^
***Newborn***			
**Gender (Boys/Girls)**	19/29	12/24	8/8
**Birth weight**	3300(3050–3587)	3400(3150–3587)	3425(3050–3600)
**25(OH)D3 (ng/mL)**	9(6–12)	17(13–19)^a***,c***^	29(20–46)^a***,b**^
**Leukocyte (10**^**3**^**/mm**^**3**^**)**	17,354 ± 4108	17,011 ± 4080	15,707 ± 2256
**Hemoglobin (g/dL)**	17.5(13.3–21.2)	13.7(10.3–19.1)^a***,c***^	16.7(14.3–18.9)^b***^
**Eosinophil (10**^**3**^**µL)**	0.48(0.02–1.90)	0.61(0.01–2.00)^c*^	0.95(0.20–1.50)^a**,b*^
**Neutrophil (10**^**3**^**/mm**^**3**^**)**	15.7(9.3–19.9)	9.8(4.9–14.3)^a***^	7.4(4.6–11.3)^a***,b**^
**Lymphocyte (10**^**3**^**/mm**^**3**^**)**	8.0(3.0–17.6)	6.6(2.1–13.4)^a*^	6.6(2.6–21.0)
**Monocyte (10**^**3**^**/mm**^**3**^**)**	7.59(1.40–16,20)	11.15(2.60–33.60)^a**^	12.96(4.40–80.70)^a*^
**Platelet (mm**^**3**^**)**	257(213–353)	255(154–360)	250(196–320)
**NLR**	2.3(1.0–6.0)	1.8(0.4–4.4)^a*,c*^	1.3(0.4–3.5)^a***,b*^
**LMR**	1.11(0,91–2.14)	0.78(0.14–2.23)^a***^	0.89(0.03–3.50)^a*^
**PLR**	39(14–92)	51(17–134)^a*^	47(14–79)
**NMR**	2.84(0.81–11.50)	1.22(0.15–5.15)^a***^	1.00(0.04–2.45)^a***^

*NLR* Neutrophil–Lymphocyte Ratio, *LMR* Lymphocyte-Monocyte Ratio, *PLR* Platelet- Lymphocyte Ratio, *NMR* Neutrophil- Monocyte Ratio

^a^vs deficient

^b^vs insufficient

^c^ vs sufficient

^*^p < 0.05, **p < 0.01, ***p < 0.001

In the deficient group, the maternal vitamin D status was found to be statistically significantly lower compared to both insufficient and sufficient group (*p* = 0.00, *p* = 0.00, respectively). Maternal vitamin D status was significantly elevated in both sufficient [35 (30–45)] and insufficient group [18 (17–19)] compared to deficient group [10 (9–11)] (*p* = 0.000). Serum vitamin D status of newborns who received up to 1200 IU vitamin D supplements per day were in the range of 30 ng/mL.

There was a statistically significant difference between the deficient, sufficient and insufficient groups in terms of maternal hemoglobin, eosinophil, leukocyte, monocytes, PLT, PLR and NMR (*p* < 0.05). However, there was no statistically significant difference between the groups in terms of maternal neutrophil, lymphocyte and NLR (*p* > 0.05).

Among the deficient, insufficient and sufficient groups, the genders and birth weight of newborns were not statistically significant. Parallel to maternal and newborn vitamin D status were also statistically different between the groups (*p* < 0.05). Moreover, there was a statistically significant difference was found between the deficient, sufficient and insufficient groups in terms of newborn hemoglobin, neutrophil, monocytes, NLR, PLT, PLR and NMR (*p* < 0.05).

**Fig. 2:**
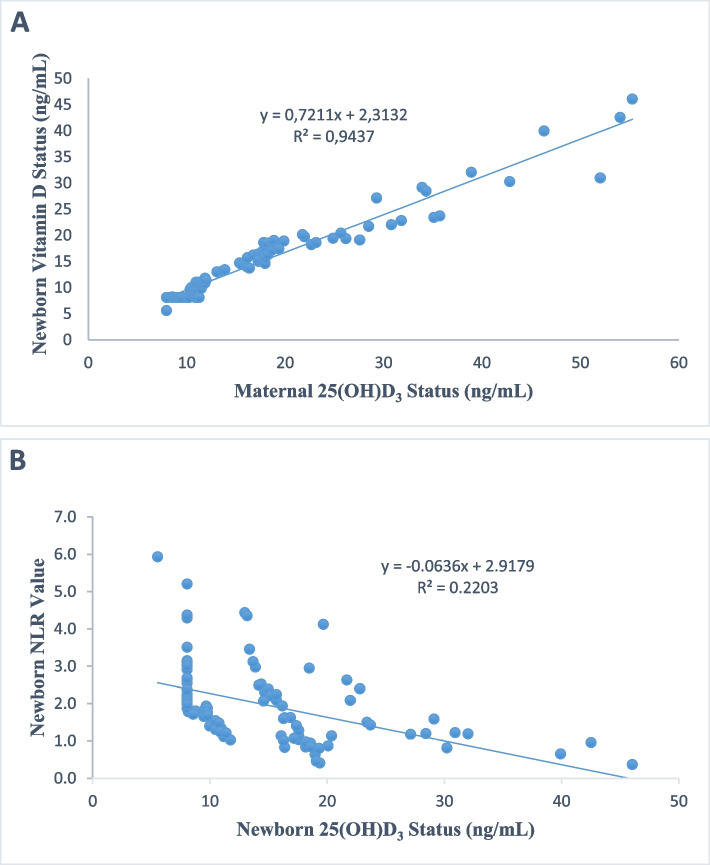
**A** Correlation analysis in between maternal and newborn vitamin D status. **B** Correlation analysis in between newborn NLR value and newborn vitamin D status

Maternal vitamin D status were very strong positive correlated with newborn vitamin D status (*r* = 0.97, *p* = 0.00) (Fig. 2 A). In addition to this correlation findings, a statistically significant moderately negative correlation was found between newborn NLR value and newborn vitamin D status (*r* = -0.61, *p* = 0.00) (Fig. 2 B).

## Discussion

Due to the fact that vitamin D deficiency or insufficiency has increased significantly all over the world in recent years, a large number of studies have been carried out on this subject. In addition to the effects of vitamin D status on bone health and muscle strength, its effects on infectious diseases are also noteworthy. In current study, NLR values were found to be significantly higher in patients with vitamin D deficiency in newborns. There was also a positive correlation between maternal and newborn vitamin D status. The newborn NLR were negative correlated with newborn vitamin D status. Our study revealed an inverse relationship between non-invasive, easy and inexpensive markers of inflammation and vitamin D status. Vitamin D deficiency may increase susceptibility to infection.

Insufficient exposure to sunlight and insufficient vitamin D stores during pregnancy and lactation are important health problems that cause insufficient vitamin D levels in both breast milk and newborn babies. It has been reported that vitamin D deficiency is seen at a very high rate in women of childbearing age [14]. In current study, in the deficient group, the maternal vitamin D status (ng/mL) was significantly lower compared to both insufficient group and sufficient group. very strong positive correlation was found between maternal serum vitamin D status and serum vitamin D status of newborn. Newborn with a low vitamin D status in their mothers are fed with breast milk containing insufficient vitamin D in the postnatal period, and Vit D deficiencies are exacerbated. This shows that maternal vitamin D deficiency should be given importance.

In a study conducted in India, the average vitamin D status of newborns (n:117) was found to be 8.4 ± 5.7 ng/mL, and 95.7% of them had a vitamin D status lower than 20 ng/mL. In the same study, they reported that there was a strong correlation between cord blood 25(OH)D levels and maternal 25(OH)D levels [15]. Sources of vitamin D in a newborn are transfer through the placenta, synthesis of vitamin D in the mother’s milk and in the skin through the rays of the sun [16]. Since the first-term vitamin D status of newborn babies are related to the vitamin D status transmitted from the mother through the placental transmission, it is important to evaluate pregnant women for vitamin D deficiency and to provide vitamin D supplementation in terms of supporting mother and baby health. Due to these high rates of vitamin D deficiency during pregnancy, various vitamin D screening and support programs have been developed. It has been demonstrated by some studies that daily 400 IU vitamin D replacement administered during pregnancy is insufficient to keep the vitamin D status in the mother and therefore the newborn at the desired level [17]. In current study, there was no statistically significant difference between the groups in maternal age, maternal socio-economic status, maternal daily sunlight exposure, delivery route. However, a significant difference was found between the vitamin D status in the newborns of pregnant women who received and did not receive vitamin D supplementation. Vitamin D status of newborns were lowest in those whose mothers did not receive vitamin D supplementation, while those who received regular vitamin D were highest. At the same time, serum vitamin D status of newborns who received up to 1200 IU vitamin D supplements per day were in the range of 30 ng/mL. In a study conducted in Spain, respiratory tract diseases are less common in the first year of babies with high vitamin D status during pregnancy. However, it does not change the prevalence of childhood asthma and wheezing [18]. The risk of type 1 DM increases in the children of pregnant women with low 25(OH)D levels during pregnancy [19]. Pregnant women are at risk of vitamin D deficiency. According to all these results, it seems that subclinical vitamin D deficiency should be emphasized and vitamin D replacement should be given to pregnant women in the last trimester. As of May 2011, Turkey-Ministry of Health has started a campaign for vitamin D replacement to pregnant and breastfeeding mothers regardless of blood level. Routine vitamin D replacement in the primary care follow-up of pregnant women will have a positive effect on the health of pregnant women and newborns. Our results are in agreement with other studies [20–25].

Serum 25-(OH)D3 levels are generally accepted as determinants of vitamin D status. Studies reveal that 1,25(OH)2 D3 functions as a cytokine and an intermediate required for production in the monocyte-macrophage system, and therefore is a potent immune-modulator [26, 27]. It is thought that vitamin D status can affect many different tissues in the body, including the hematopoietic system. The relationship between vitamin D status and hemogram parameters/hematological indices, which are indicators of the hematopoietic system were investigated. NLR has been used as a marker of systemic inflammation in recent years. It is thought that monocytes and macrophages can reduce an immune response when serum vitamin D status fall below 20 ng/mL. NLR has been used as a systemic inflammation marker in recent years. In current study, it was found that there is a relationship between vitamin D status of newborns and NLR. The newborn NLR were negative correlated with newborn vitamin D status. Moreover, there was a statistically significant difference was found between the deficient, sufficient and insufficient groups in terms of newborn hemoglobin, neutrophil, monocytes, NLR, PLT, PLR and NMR. This result shows that, the vitamin D status may be associated with inflammation, and a decrease in vitamin D may cause an increase in NLR but more studies needs to be done for further pathway explanations. A correlation was found between decreased serum 25-(OH) D3 and increased NLR in mothers of preterm infants. No relationship was observed between birth weight, maternal age, socio-economic status, daily sunlight exposure, intake vitamin D supplementation during pregnancy with NLR and other hematologic indices. These results show that vitamin D and NLR play a role in the etiology of spontaneous preterm birth [28]. Vitamin D deficiency may increase susceptibility to infection. Vitamin D deficiency may play a role in the pathogenesis of inflammation and low serum vitamin D status may lead to infectious diseases [29–32]. Detection of low NLR rate with a non-invasive, inexpensive and easily obtainable test such as CBC may warn us about vitamin D deficiency after other pathologies are excluded. Of both markers, NLR, a combination of circulating neutrophil and lymphocyte counts, is a representative index of systemic inflammation in newborns.

The best source that can meet the need for vitamin D in the body is the sun. Deficiency during pregnancy can be a risk for both mother and baby. Although vitamin D supplementation can be compensated in studies conducted to date, there is not enough information about its safety. It is definitely important to give vitamin D supplementation in the later stages of pregnancy for people living in places where the winter season is long. In this retrospective case–control study, maternal vitamin D deficiency is still a serious problem, based on newborn vitamin D status. For this reason, it is necessary to strengthen and expand the measures to prevent and eliminate maternal vitamin D deficiency. According to the results of our study, we think that vitamin D status in mothers should be examined before pregnancy is planned and treatment should be started in case of deficiency, and if it is not possible, it should be evaluated as one of the routine control parameters during pregnancy. We especially recommend that pregnant women with vitamin D < 20 ng/mL take vitamin D. Timely diagnosis and treatment of mother’s vitamin D deficiency can prevent vitamin D deficiency that may develop in newborns. Adequate vitamin D intake should be provided to expectant mothers, pregnant women, mothers during breastfeeding, and all babies.

## Conclusions

Increased NLR may be the indicator of underlying serious vitamin D deficiency in newborns. Because vitamin D deficiency may increase susceptibility to infection, physicians should be alert and order a vitamin D assay in patients with high NLR, PLT, PLR and NMR especially in areas where vitamin D deficiency is endemic. Thus, possible inflammation can be prevented upon following appropriate treatment. In order to prevent vitamin D deficiency and insufficient in both mother and baby, additional vitamin D supplements should be prescribed to pregnant women during pregnancy. As a conclusion, our findings support the association of vitamin D and inflammation in newborn. The evaluation of these parameters in a larger population may help further confirm these findings.

## Data Availability

Participant-level data are available from the corresponding author.

## Abbreviations

Vit D: Vitamin D, 25(OH)D3
T1DM: Type 1 DM
NLR: Neutrophil to lymphocyte ratio
LMR: Lymphocyte to monocyte ratio
PLR: Platelet to lymphocyte ratio
CBC: Complete blood count
MCV: Mean corpuscular volume
CRP: C-reactive protein
ESR: Erythrocyte sedimentation rate
MPV: Mean platelet volume
